# Misfolded protein deposits in Parkinson’s disease and Parkinson’s disease-related cognitive impairment, a [^11^C]PBB3 study

**DOI:** 10.1038/s41531-024-00708-z

**Published:** 2024-05-03

**Authors:** Michele Matarazzo, Alexandra Pérez-Soriano, Nasim Vafai, Elham Shahinfard, Kevin Ju-Chieh Cheng, Jessamyn McKenzie, Nicole Neilson, Qing Miao, Paul Schaffer, Hitoshi Shinotoh, Jeffrey H. Kordower, Vesna Sossi, A. Jon Stoessl

**Affiliations:** 1 Djavad Mowafaghian Centre for Brain Health, Pacific Parkinson’s Research Centre, University of British Columbia & Vancouver Coastal Health, Vancouver, BC Canada; 2 HM CINAC, Hospital Universitario HM Puerta del Sur, Móstoles, Madrid, Spain; 3 Department of Medicine, Faculty of Medicine, Complutense University of Madrid, Madrid, Spain; 4 Department of Physics and Astronomy, University of British Columbia, Vancouver, BC Canada; 5 TRIUMF, Vancouver, BC Canada; 6 Department of Functional Brain Imaging Research, National Institute of Radiological Sciences, Chiba, Japan; 7 ASU-Banner Neurodegenerative Disease Research Center and School of Life Sciences, Arizona State University, Tempe, AZ USA; 8 Division of Neurology, Department of Medicine, University of British Columbia, Vancouver, BC Canada

**Keywords:** Parkinson's disease, Translational research

## Abstract

Parkinson’s disease (PD) is associated with aggregation of misfolded α-synuclein and other proteins, including tau. We designed a cross-sectional study to quantify the brain binding of [^11^C]PBB3 (a ligand known to bind to misfolded tau and possibly α-synuclein) as a proxy of misfolded protein aggregation in Parkinson’s disease (PD) subjects with and without cognitive impairment and healthy controls (HC). In this cross-sectional study, nineteen cognitively normal PD subjects (CN-PD), thirteen cognitively impaired PD subjects (CI-PD) and ten HC underwent [^11^C]PBB3 PET. A subset of the PD subjects also underwent PET imaging with [^11^C](+)DTBZ to assess dopaminergic denervation and [^11^C]PBR28 to assess neuroinflammation. Compared to HC, PD subjects showed higher [^11^C]PBB3 binding in the posterior putamen but not the substantia nigra. There was no relationship across subjects between [^11^C]PBB3 and [^11^C]PBR28 binding in nigrostriatal regions. [^11^C]PBB3 binding was increased in the anterior cingulate in CI-PD compared to CN-PD and HC, and there was an inverse correlation between cognitive scores and [^11^C]PBB3 binding in this region across all PD subjects. Our results support a primary role of abnormal protein deposition localized to the posterior putamen in PD. This suggests that striatal axonal terminals are preferentially involved in the pathophysiology of PD. Furthermore, our findings suggest that anterior cingulate pathology might represent a significant in vivo marker of cognitive impairment in PD, in agreement with previous neuropathological studies.

## Introduction

Most sporadic neurodegenerative diseases are characterized by the presence of misfolded proteins aggregated in different brain regions, including both intra- and extra-cellular deposition. Although the role of these proteins is still debated, their location often correlates with neuronal loss. In Parkinson’s disease (PD) there is evidence of Lewy pathology (LP), including Lewy bodies (LB) in neuronal somata and Lewy neurites (LN), located in axons. The origin and role of LP in PD are still largely unknown, but its presence and location are included in PD neuropathological diagnostic criteria^1^ and the most widely used staging method^2^. Following the discovery of familial forms of PD related to pathogenic variants in *SNCA*, the gene encoding α-synuclein (α-syn), this protein has been recognized as an essential component of LP^3^. Importantly, it is common to find mixed pathology in PD, with tau and β-amyloid aggregates, which are more common in advanced stages and in patients with cognitive involvement^4^.

Despite vast research efforts, the role of protein aggregates and how they contribute to neurodegeneration is one of the main unanswered questions in PD, and even the possibility that they are protective rather than pathogenic is still debated. Interestingly, protein deposits are observed from very early and even prodromal stages of the disease^5^.

The lack of tools to assess neuropathology in vivo is a major limitation in PD, as this would be important to understand the disease and its pathophysiology, as well as providing a biomarker to assist in diagnosis and assessment of disease-modifying therapies.

The positron emission tomography (PET) tracer [^11^C]PBB3 binds to misfolded tau protein, with sensitivity to 3-repeat and 4-repeat isoforms. Unlike other first generation (and several 2^nd^ generation) tau ligands, [^11^C]PBB3 does not appear to have off-target binding to monoamine oxidase, neuromelanin or iron. Furthermore, in previous work we have shown PBB3 binding in pre-symptomatic *SNCA* multiplication carriers and a single MSA patient^6^, consistent with the known topography of α-syn pathology. Post-mortem fluorescence and autoradiographic evidence confirmed binding of PBB3 to α-syn in Lewy body diseases and MSA, even in the absence of tau pathology^7^. We thus hypothesized that [^11^C]PBB3 could label a range of misfolded protein pathology in idiopathic PD. We evaluated the presence of presumed protein misfolding in vivo in a cohort of cognitively normal PD patients (CN-PD) and compared the binding with healthy controls. We also evaluated the distribution of misfolded protein in cognitively impaired PD (CI-PD) patients and assessed the relationship with cognition. As protein misfolding in neurodegenerative disorders may be associated with and modulated by neuroinflammatory responses^4^, a subset of participants underwent a scan with the 18 kDa translocator protein (TSPO) marker [^11^C]PBR28. This served the additional purpose of ruling out the possibility that increased [^11^C]PBB3 signal was due to neuroinflammation, given that other first generation tau tracers bind to monoamine oxidase B expressed in glial cells.

## Results

### Baseline characteristics

MoCA and BDI scores were not available for one CI-PD subject, DRS-2 scores were not available for six CI-PD subjects. MDS-UPDRS was not available for two CI-PD subjects. These scores were not available because the patients were either unable or unwilling to complete the tests. Both PD groups were typical of the broader PD population and differed from one another on the basis of cognition only. Baseline age and sex did not show significant differences between groups. MDS-UPDRS III score was higher in both PD groups compared to HC and similar between the two groups (Table 1). MoCA scores were lower in CI-PD, as expected. CN-PD MoCA score was not different from HC group. CI-PD had a higher BDI score compared to HC, but the mean value was below the cut-off for depression. The median age was 68.2 in HC, 68.6 in CN-PD and 71.7 in CI-PD, and median disease duration was 7.8 years in CN-PD and 11.9 in CI-PD, and these were not significantly different from one another.

**Table 1:** Baseline characteristics and comparisons

	Group			*p* value			
	Healthy controls (*n* = 10)	CN-PD (*n* = 19)	CI-PD (*n* = 13)	Overall	HC vs CN-PD	HC vs CI-PD	CN-PD vs CI-PD
Age	68.18 [58.05, 71.82]	68.62 [65.76, 72.03]	71.70 [68.94, 74.29]	0.351	0.646	0.239	0.212
Sex (female)	4 (40.0)	4 (21.1)	4 (30.8)	0.510	0.390	0.685	0.684
MoCA	27.50 [26.25, 28.75]	28.00 [26.50, 28.50]	20.50 [18.75, 23.00] (*n* = 12)	**<0.001**	0.705	**<0.001**	**<0.001**
BDI	2.50 [0.25, 8.50]	5.00 [2.00, 8.00]	8.50 [5.50, 14.25] (*n* = 12)	0.064	0.368	**0.040**	0.061
Disease duration	–	7.76 [3.97, 9.64]	11.91 [6.08, 15.54]	0.081	NA	NA	0.081
MDS-UPDRS III	5.00 [2.00, 9.00]	22.00 [18.00, 32.50]	29.00 [23.00, 41.50] (*n* = 11)	**<0.001**	**<0.001**	**<0.001**	0.081

Quantitative variables are represented as “median [IQR]” and qualitative ones as “n (%)” unless otherwise specified.

*BDI* Beck Depression Inventory, *CI-PD* Cognitively impaired Parkinson’s disease subjects, *CN-PD* Cognitively normal Parkinson’s disease subjects, *MDS-UPDRS III* Movement Disorder Society-sponsored revision of the Unified Parkinson’s Disease Rating Scale part III, *MoCA* Montreal Cognitive Assessment test.

### PBB3 binding in dopaminergic pathway ROIs

**Fig. 1:**
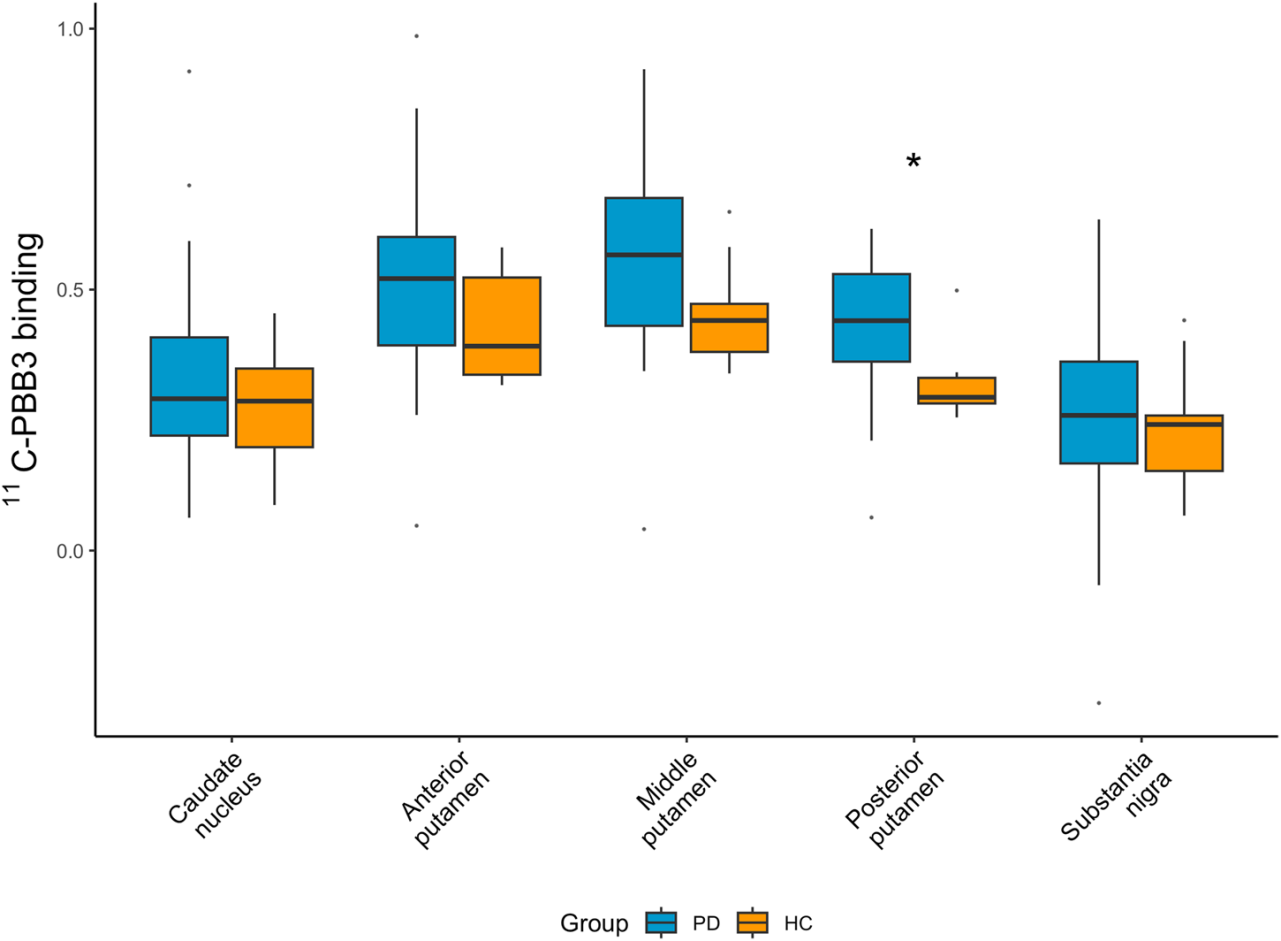
[^11^C]PBB3 binding in the nigrostriatal regions. [^11^C]PBB3 binding in the nigrostriatal pathway in healthy controls and PD subjects. Data are depicted as boxplots (center line—median, box limits—upper and lower quartiles, whiskers—1.5× interquartile range) *p < 0.05, corrected for multiple comparisons. Groups were compared using Mann–Whitney *U* test.

**Fig. 2:**
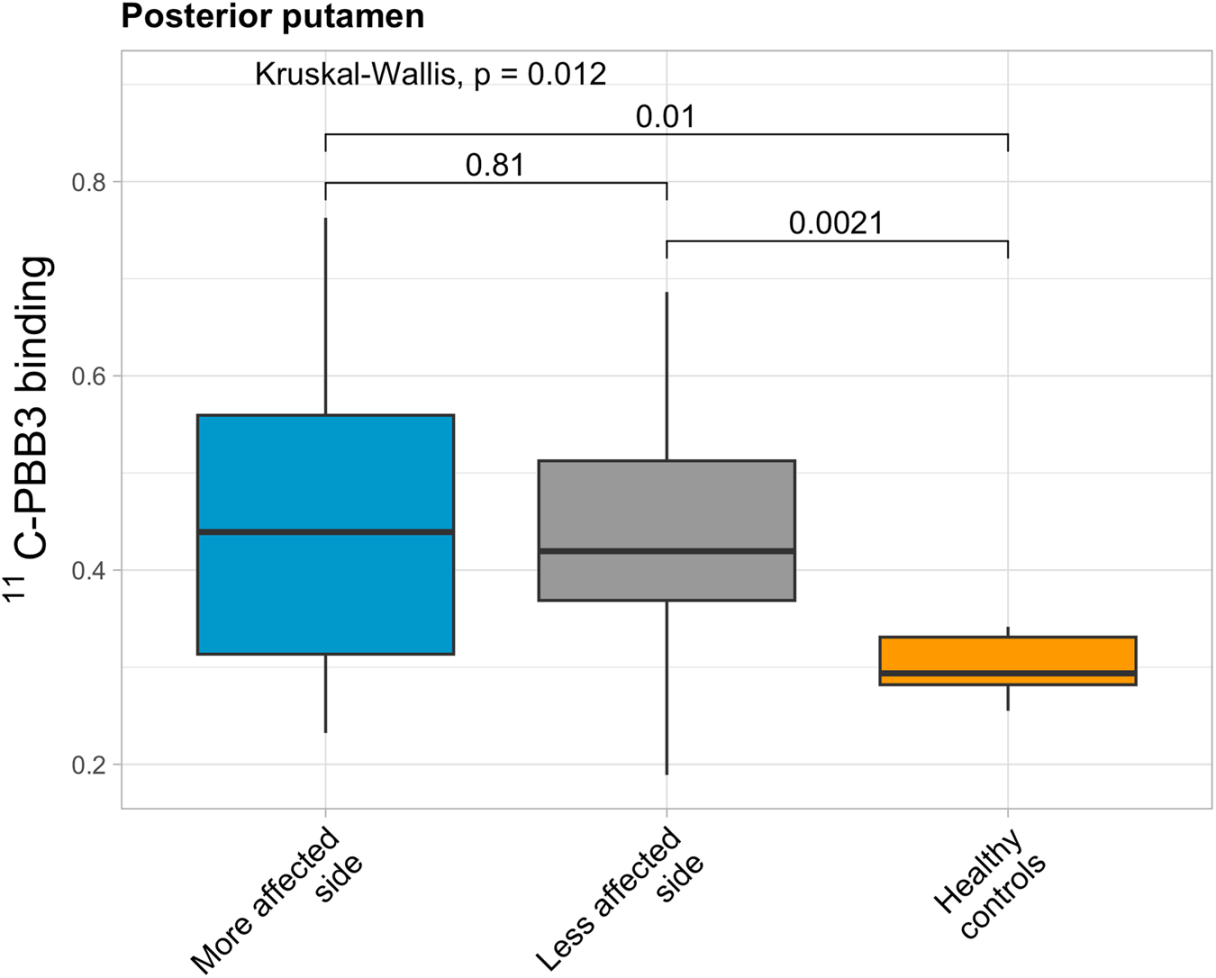
[^11^C]PBB3 binding in the posterior putamen. [^11^C]PBB3 binding in the posterior putamen in healthy controls and in more and less affected hemispheres in PD subjects. Data are depicted as boxplots (center line—median, box limits—upper and lower quartiles, whiskers—1.5× interquartile range).

Average [^11^C]PBB3 binding was higher in PD compared to HC in the posterior putamen (Fig. 1) (corrected *p* = 0.02), and this was seen in both more and less affected hemispheres (Fig. 2). Of note, median nigral binding was marginally increased, but not statistically different from HC. Results were similar when comparing HC with only the CN-PD sample (Supplementary Table 1).

There was no significant correlation between disease duration and [^11^C]PBB3 binding, whether or not age was added as a covariate. There was no significant correlation between [^11^C]PBB3 and [^11^C](+)DTBZ binding in any of the striatal regions.

In the linear regression analysis, there was no significant correlation between [^11^C]PBB3 binding and age in either group in striatal ROIs.

### PBB3 binding in other ROIs

**Fig. 3:**
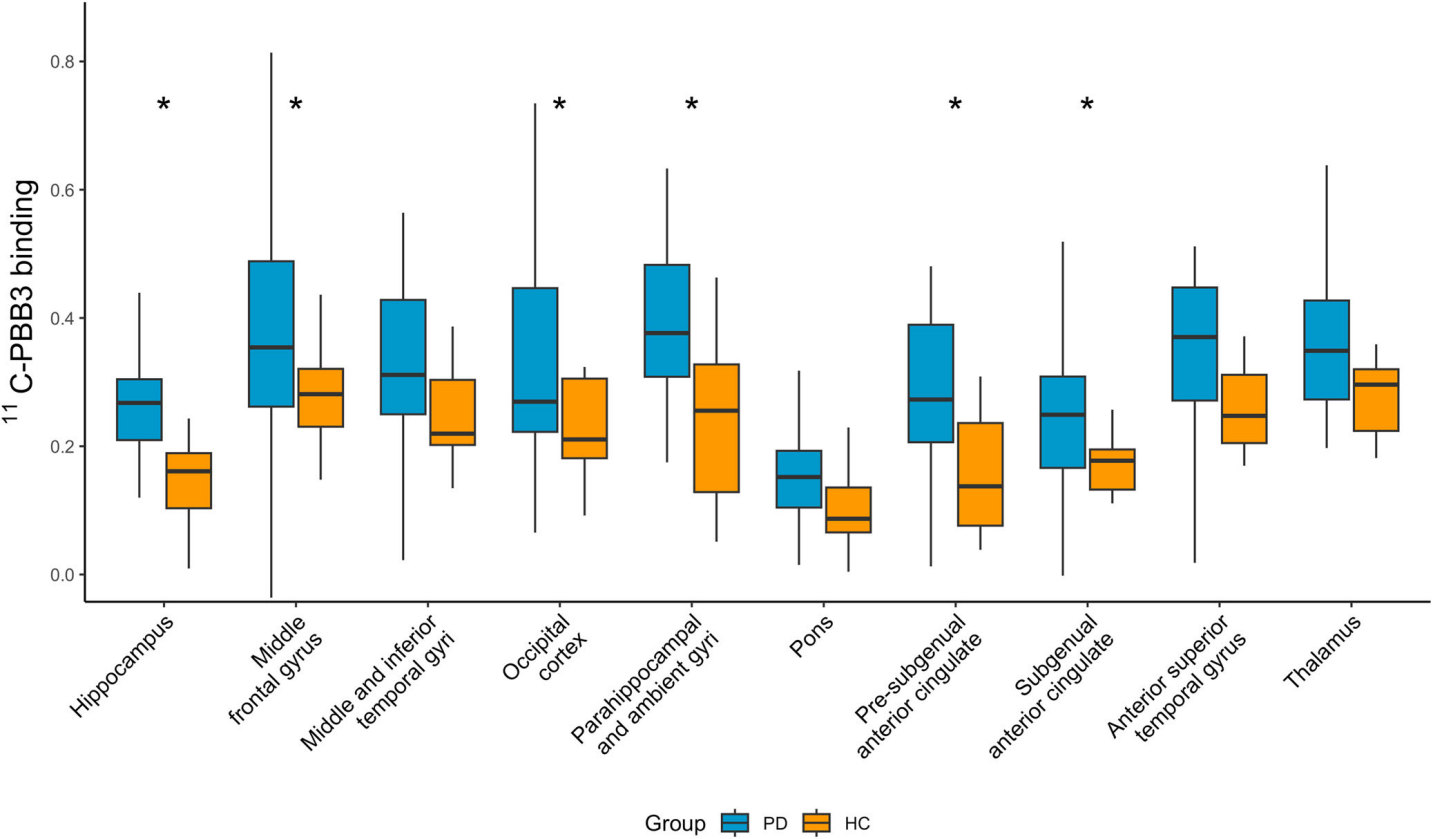
[^11^C]PBB3 binding in other PD-relevant regions. Exploratory analysis of [^11^C]PBB3 binding in secondary outcome ROIs in healthy controls and PD subjects. Data are depicted as boxplots (center line—median, box limits—upper and lower quartiles, whiskers—1.5× interquartile range). *p < 0.05, non-corrected for multiple comparisons. Groups were compared using Mann–Whitney *U* test.

The exploratory analysis of secondary brain regions showed higher [^11^C]PBB3 BP_ND_ in PD than HC in most regions, including hippocampus (nominal *p* = 0.01), entorhinal/parahippocampal cortex (nominal *p* = 0.02), subgenual and presubgenual anterior cingulate (nominal *p* = 0.02) (Fig. 3), however these differences did not survive multiple comparison correction.

### PBB3 binding and cognition

[^11^C]PBB3 BP_ND_ was significantly higher in CI-PD compared with HC in the subgenual and pre-subgenual anterior cingulate cortex (corrected *p* = 0.040 and 0.005, respectively). Binding in pre-subgenual anterior cingulate was higher in CI-PD compared to CN-PD (corrected *p* = 0.012). Other cortical regions also showed higher values in CI-PD, but differences were not significant following correction for multiple comparisons (Table 2).

**Table 2:** Comparison of [^11^C] PBB3 binding in cognitively intact and cognitively impaired PD

	Group		
	CN-PD (*n* = 19)	CI-PD (*n* = 13)	Multiple comparison- adjusted *p* value
Substantia nigra	0.29 [0.20, 0.34]	0.19 [0.12, 0.38]	NS
Caudate	0.28 [0.19, 0.36]	0.31 [0.23, 0.49]	NS
Anterior putamen	0.48 [0.39, 0.54]	0.60 [0.50, 0.66]	NS
Middle putamen	0.55 [0.42, 0.67]	0.58 [0.44, 0.70]	NS
Posterior putamen	0.44 [0.36, 0.52]	0.44 [0.37, 0.54]	NS
Hippocampus	0.28 [0.25, 0.29]	0.20 [0.12, 0.33]	NS
Middle frontal gyrus	0.34 [0.26, 0.43]	0.41 [0.34, 0.67]	NS
Middle and inferior temporal gyrus	0.30 [0.24, 0.35]	0.37 [0.28, 0.43]	NS
Occipital cortex	0.26 [0.23, 0.35]	0.38 [0.22, 0.61]	NS
Parahippocampal ambient gyri	0.36 [0.31, 0.42]	0.48 [0.35, 0.58]	NS
Presubgenual anterior cingulate	0.21 [0.13, 0.26]	0.38 [0.30, 0.47]	0.012
Pons	0.15 [0.11, 0.17]	0.17 [0.10, 0.21]	NS
Subgenual anterior cingulate	0.21 [0.16, 0.27]	0.30 [0.20, 0.39]	NS
Subcallosal	0.20 [0.17, 0.31]	0.37 [0.20, 0.47]	NS
Superior anterior temporal gyrus	0.30 [0.28, 0.40]	0.45 [0.22, 0.50]	NS
Thalamus	0.35 [0.29, 0.40]	0.31 [0.24, 0.48]	NS
Amygdala	0.20 [0.16, 0.27]	0.27 [0.23, 0.34]	NS

Variables are represented as “median [IQR]”.

*NS* non-significant (i.e., *p* value adjusted for multiple comparisons >0.05).

**Fig. 4:**
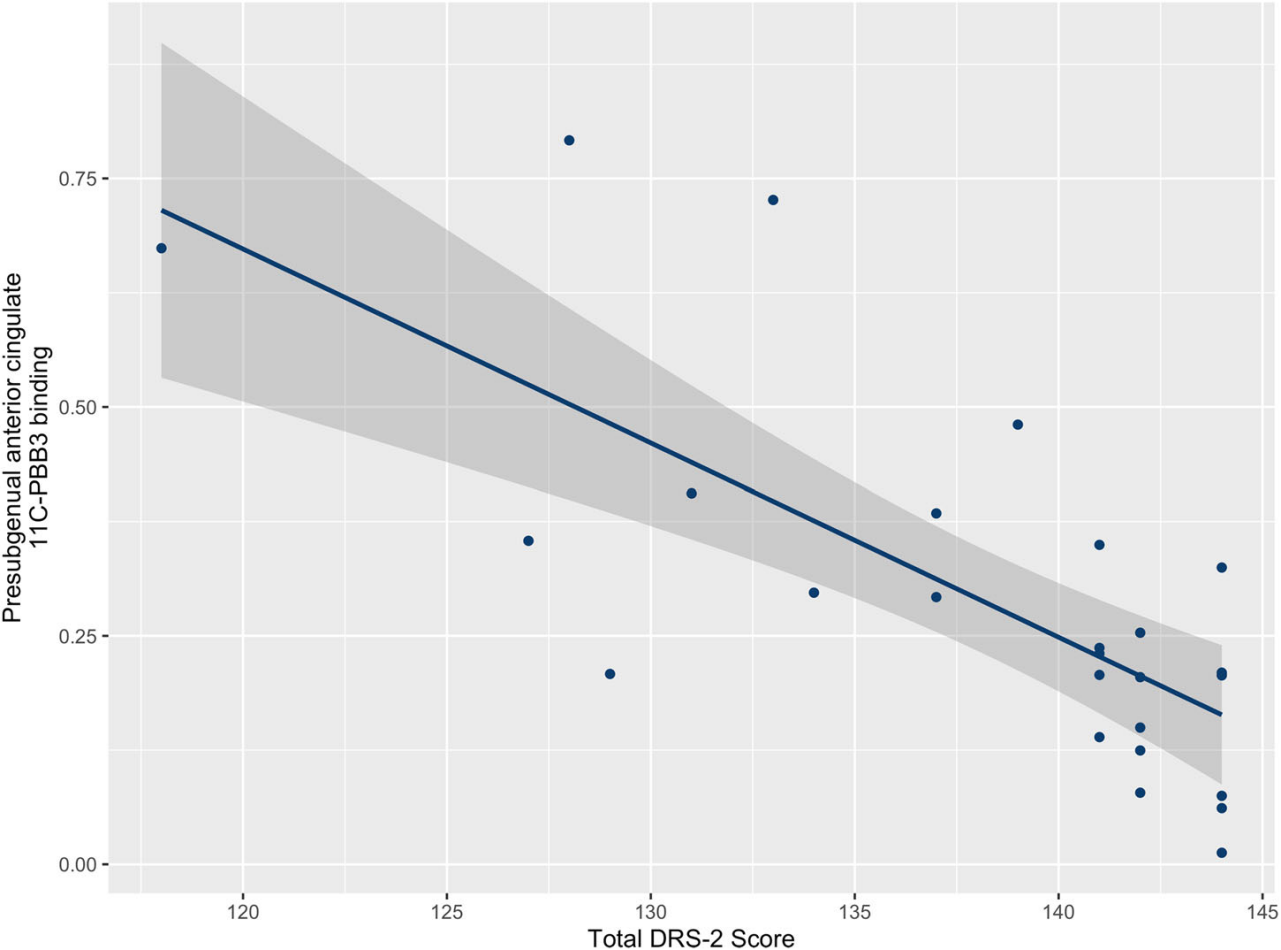
[^11^C]PBB3 binding in cortical regions correlates with cognitive outcomes. Correlation between [^11^C]PBB3 binding in pre-subgenual anterior cingulate cortex and total DRS-2 score in PD subjects. DRS-2, Dementia rating scale 2. *P* value corrected for multiple comparisons.

Across all PD subjects (CI- and CN-PD combined), we found an inverse correlation between the DRS-2 score and [^11^C]PBB3 BP_ND_ in the pre-subgenual anterior cingulate (Spearman rho = −0.73, *p* < 0.001) (Fig. 4). This finding persisted in a linear regression analysis following adjustment for age and BDI to account for the possible confound of mood changes or age-related cognitive decline (*p* = 0.003).

### PBB3 and PBR28 binding correlation

There was no relationship between [^11^C]PBB3 and [^11^C]PBR28 binding across subjects (*n* = 6) in any of the studied regions. In most individuals, the within-subject ranked correlation (i.e., correlation of the two tracers in each of the 13 regions for each given participant) showed no relationship between [^11^C]PBB3 and [^11^C]PBR28 binding, except for two participants with early disease (Supplementary Table 2). The individual within-subject ranked correlation coefficients relating [^11^C]PBB3 and [^11^C]PBR28 binding were inversely related to disease duration (Spearman’s rho = −0.86, *p* = 0.028, *n* = 6, Supplementary Fig. 1). This result was driven by the two participants with early disease, raising the possibility that abnormal protein deposition may be associated with neuroinflammation in early disease only. The additional inclusion of the medium affinity binders (one early PD, two advanced PD) did not change the results (Spearman’s rho = −0.68, *p* = 0.043, *n* = 9). Taken together, these findings mean it is unlikely that [^11^C]PBB3 is binding off target to inflammatory or glial cells.

## Discussion

Our data show increased [^11^C]PBB3 binding in bilateral posterior putamen of PD patients. Based on previous in vivo^6^ and post-mortem^7^ findings, we believe this likely reflects a relatively greater neuropathological burden of abnormal protein aggregation at this level in PD. We did not find significant increases in other regions of the striatum, nor in the substantia nigra. These findings are compatible with the hypothesis that striatal nerve terminals are the main target of misfolded proteins in PD^8^. Of note, on the assumption (see below) that [^11^C]PBB3 may bind to misfolded α-syn in addition to tau, at least some of the [^11^C]PBB3 binding detected could be due to extraneuronal α-syn, which has been demonstrated in several brain regions in PD, including the striatum. This is particularly relevant because most dopaminergic striatal innervation has disappeared at the point the scans were taken^9^. We did not find a correlation of [^11^C]PBB3 BP_ND_ with either disease duration or the degree of dopaminergic denervation in any of the nigrostriatal ROIs. We believe this may reflect a combination of abnormal protein deposition, counterbalanced by progressive neuronal loss. Indeed, these in vivo imaging data are supported by the demonstration of early increases in aggregated α-syn in the putamen of patients with minimal motor features and suspected prodromal PD, who show greater striatal LN pathology than advanced PD, suggesting early involvement of nerve terminals^10^. Despite PD frequently manifesting as an asymmetric condition and the asymmetrical pattern of dopamine denervation, the asymmetry of other neuropathological features, such as protein aggregation, has not been documented, and therefore, the lack of asymmetric [^11^C]PBB3 uptake in the putamen of patients with PD of moderate disease duration may not be entirely surprising. Nevertheless, we did observe a trend towards higher values on the more affected side, with non-significant higher median values in both putamen (anterior, middle, posterior and whole) and SN. This difference between sides is small in magnitude and the variance is high, but this trend suggests the need for further investigation to elucidate the potential asymmetrical involvement of neuropathological features in PD beyond dopamine denervation.

The site of initial protein aggregation and neurodegeneration within the nigrostriatal pathway is unknown. However, in the widely held theory of stereotypic caudal-to-rostral disease progression, the SN is thought to be affected prior to subsequent loss of striatal terminals^2^. Indeed, a recent modification of this hypothesis does not consider the role of pathology starting in striatal nerve terminals^11,12^. Alternative hypotheses are based on several relevant findings. First, the motor manifestations of PD almost always show an asymmetrical focal onset and a somatotopic pattern of spread; therefore, it would be logical to think that the brain region of initial involvement might also have a somatotopic organization. The lack of known SN somatotopy in humans as well as in other primates^13^ and the diffuse axonal arborization of the nigrostriatal neurons in the striatum, where a selective pattern of denervation is observed, cannot explain the focality of the clinical findings. In contrast, other PD-relevant structures such as the putamen and motor cortex do show somatotopic organization^14^, and therefore represent potential candidate locations where neurodegeneration may begin. Secondly, axonal transport dysfunction has been shown to represent an early and crucial pathological mechanism in PD, and may precede degeneration^15,16^. This finding has also been reproduced in vitro in rat cultured neurons transfected with plasmids containing human mutant α-syn, suggesting a critical role in perikaryal accumulation of α-syn^17^. Indeed, axonal transport defects are seen only in those PD nigral neurons that contain Lewy pathology, while adjacent neurons without Lewy pathology demonstrate normal levels of axonal transport motors, findings that are duplicated in synuclein-based PD models^16^. Thirdly, α-syn is physiologically located mainly in axonal terminals, and therefore it is likely that the pathogenic changes and aggregation start at this level, as indeed supported by the observations that LN precede the appearance of LB in most affected brain structures in PD^2^. In the peripheral nervous system, the axonal aggregation of α-syn is more abundant and precedes the involvement of ganglia, suggesting a centripetal axon-to-soma progression^18^. Also, in non-human primate models, α-syn is transported from axon terminals to neuronal bodies, with LB-like aggregation in the SN following intraputaminal α-syn injection^19^, and selective increase of α-syn in the posterior putamen following enteric injection of PD-derived α-syn, supporting the relevance of posterior putamen as a key target for α-syn pathology^20^. Finally, both neuropathological and molecular imaging evidence have shown that at the time of diagnosis the striatal axonal loss exceeds neuronal loss in the SN, supporting the early involvement of striatal terminals in the pathophysiology of PD^9,21,22^. Interestingly, the bottom-up and top-down hypotheses may not be exclusionary, and there might be a different pattern of progression in different phenotypes of PD patients.

Off-target binding to monoamine oxidase B (MAO-B) has been demonstrated for several tau tracers^23^. MAO-B is mostly located in glial cells, and its expression in reactive astrocytes has been shown in several diseases^24^. To date there is no evidence of PBB3 binding to MAO. Nevertheless, we scanned a subset of PD patients with [^11^C]PBR28, a marker of microglial activation, and found no relationship to [^11^C]PBB3 binding in those regions with increased PBB3 binding, consistent with a lack of off-target binding to MAO. Of note, the within-individual Spearman rho correlation coefficient between the ranked binding of the two tracers across different brain regions decreased significantly with increased disease duration. While based on a very limited number of subjects, this suggests that the two neuropathological processes (i.e., neuroinflammation and protein aggregation) might occur in parallel in the early stages of the disease, but less so when disease is more advanced. Previous pre-clinical studies have shown that abnormal protein deposits may have a pro-inflammatory effect in PD^25,26^, while other studies have provided evidence of microglial activation promoting protein aggregation^27^, suggesting a complex interplay between protein aggregation and inflammation. Future studies investigating the longitudinal progression of neuroinflammation in different brain regions in PD will provide further insights into its role in neurodegeneration and its neurobiological relationship with dopamine denervation and protein aggregation.

As noted above, unlike other 1^st^ generation tau PET ligands, [^11^C]PBB3 is not thought to bind to MAO and this is in keeping with the overall lack of relationship to [^11^C]PBR28 binding in the current study. The binding of [^11^C]PBB3 to misfolded tau has been extensively characterized both in vitro and in vivo and is shown to bind to both 3R/4R and 4R tau pathology^6,28,29^. Both post-mortem^7^ and in vivo^6^ studies suggest that PBB3 binds as well to α-syn, although the degree to which the [^11^C] tracer does so in conditions of relatively low target availability is unclear. In favor of the hypothesis that [^11^C]PBB3 binding we observe here is to α-syn and not tau is the fact that 2^nd^ generation tau tracers [^18^F]PI-2620^30^ and [^11^C]florzolotau^31^ do not show significant uptake in patients with PD compared to HC. These tracers were not available at the time this study was conducted. There was uptake of [^18^F]-AV1451 in the putamen of PD patients in a recent study, but this was attributed by the authors to off-target binding^32^. However, even if elevated [^11^C]PBB3 is indeed representative of tau binding, this might not be surprising in view of recent evidence for early tau pathology in the nigrostriatal system of subjects with mild motor deficits insufficient to be diagnosed with PD, even without α-syn aggregates^33^, nor would this possibility have any impact on our interpretation that the pathology of PD starts in nerve terminals.

Although it did not reach statistical significance following correction for multiple comparisons, [^11^C]PBB3 binding in the hippocampus and parahippocamal/entorhinal regions showed a nominal increase in binding in PD participants. Interestingly, these regions are affected early by LP, and are known to show β-amyloid and tau aggregation later in the progression of the disease^2^. More specifically, LN are abundant in the anteromedial temporal mesocortex from early stages of the disease as this represents one of the first affected sites in the cortex.

Both α-syn and tau are increased in cortical regions in PD-related cognitive impairment. Anterior cingulate, temporal, and entorhinal cortex have often been implicated as some of the primary areas affected in PD-related dementia and where pathology is negatively correlated with cognitive outcomes^34,35^. Our data mirror these neuropathological studies, with increased [^11^C]PBB3 binding in CI-PD in the anterior cingulate, where we also found a negative correlation with the cognitive outcome, despite a relatively large number of missing DRS-2 scores in the CI-PD population. Previous neuropathological studies have shown that different misfolded protein deposits follow patterned topographical distributions in the brains of people with PD, with α-syn being more abundant in the cingulate cortex, while pathology in the hippocampus and entorhinal cortex is mixed, with higher concentration of neurofibrillary tangles^35^. Consistent with this, the region most significantly related to cognitive impairment in this study was the anterior cingulate, favouring the hypothesis that, in this subject population, increased anterior cingulate [^11^C]PBB3 binding may be due to α-syn pathology, while in other regions it could represent mixed or tau aggregation. Interestingly, there was a nominal trend towards increased binding in the entorhinal/parahippocampal cortex (nominal *p* = 0.011), middle frontal gyrus (nominal *p* = 0.030), occipital cortex (nominal *p* = 0.041), subcallosal gyrus (nominal *p* = 0.041) and amygdala (nominal *p* = 0.047) in CI-PD vs. HC, but these differences did not survive multiple comparison correction. We believe that an increased sample size would allow the detection of more regions with increased protein load in the setting of CI-PD. In further support of our view that PBB3 binding in anterior cingulate reflects binding to α-syn rather than tau pathology, a recent study with [^18^F]AV1451 failed to demonstrate binding in the cingulate cortex of PD patients at high risk of dementia^32^.

Several limitations should be considered when interpreting the results. First, the signal-to-noise ratio of [^11^C]PBB3 is not ideal, which may reduce the likelihood of finding significant results. Despite that and the relatively small sample size, we found statistically significant differences, with high biological coherence and relevance. The sample size was limited by challenges with recruitment, exacerbated by the COVID-19 pandemic, as well as difficulty with tracer production. Logistical challenges as well as limited access to tracer (which has a short half-life) meant that the study was conducted in a single center. It is possible that some comparisons that failed to be statistically significant when corrected for multiple tests would have been significant with higher sample size or if the tracer were more sensitive. The [^11^C]PBR28 PET sample size was even smaller, and therefore the results should be interpreted with caution. A number of CI-PD participants did not complete the DRS-2 (*n* = 6/13), this might have had an impact on the correlation analysis, more likely reducing the statistical power to find other potential relationships that did not reach statistical significance. Secondly, disease-related atrophy of the basal ganglia and cortex may artefactually reduce the BP_ND_ in PD. To limit the impact of this artifact, we used partial-volume correction, which should at least partly address this issue.

As discussed above, [^11^C]PBB3 may bind to α-syn deposits in addition to the known tau affinity. While lack of selectivity is often not ideal for a molecular imaging tracer, it may be advantageous in this situation, as it may allow the detection of abnormal aggregation of either protein, especially considering that tracers selectively binding to α-syn are not currently available. It is indeed possible that the binding shown in the present study is related to α-syn in some regions and to tau in others. In the case of the putamen, the absence of significant binding increase in CN-PD patients with other tau tracers, including the fluorinated derivative of [^11^C]PBB3, [^18^F]PM-PBB3^36^, which has a higher tau selectivity and higher signal-to-noise ratio, lends support to our interpretation that our findings reflect α-syn rather than tau binding, although this should be confirmed in further studies. Nevertheless, a tracer with higher specificity to α-syn remains desirable. Indeed, a tracer with higher selectivity and signal-to-noise ratio would represent a major step-forward as a biomarker in the setting of clinical trials for disease-modifying therapies in PD. Recently identified derivatives of PBB3 and other compounds have shown higher α-syn selectivity in vitro and in vivo in animal models^37,38^, and show increased binding in MSA patients, in brain areas known to have high α-syn load^39^, but with limited binding in PD^40^.

Our findings demonstrate increased protein aggregation in vivo in the posterior putamen of PD patients, that may at least partially reflect α-syn pathology. The greater increases in nigrostriatal nerve terminals and other locations selectively affected by non-LB α-syn aggregation support the theory that the disease affects nerve terminals preferentially, rather than neuronal bodies. Our findings also support the role of protein misfolding in the anterior cingulate as an important determinant of cognitive function in PD.

## Methods

### Participants

Between March 2015 and January 2022, nineteen CN-PD patients and thirteen CI-PD were recruited in a cross-sectional study. Inclusion criteria were: (i) PD clinical diagnosis; (ii) age 19 years or older. Participants were recruited via the outpatient movement disorders clinic at the Djavad Mowafaghian Center for Brain Health - University of British Columbia. PD subjects who had developed cognitive impairment (at least 1 year after the PD diagnosis) or with Montreal Cognitive Assessment (MoCA) score ≤25 were classified as CI-PD. A healthy control (HC) group consisting of ten age-matched healthy participants was also recruited through electronic advertisement on the website of our institution and through printed posters. Main exclusion criteria for both groups were: (i) diagnosis of another central nervous system condition, including evidence of atypical parkinsonism, (ii) history of traumatic brain injury, (iii) any contraindication for PET and/or MRI scan. Subjects with a MoCA score ≤25 and/or a family history of movement disorders or dementia were excluded from the HC group. Other than diabetes in a single subject with CI-PD, there were no significant co-morbidities such as uncontrolled hypertension, diabetes, malignancy or chronic inflammatory disease. All participants provided written informed consent and the study was approved by the University of British Columbia Clinical Research Ethics Board. As there was no prior experience with the use of [^11^C]PBB3 in PD, a formal sample size calculation was not performed. Further recruitment at later stages was limited by the COVID pandemic as well as difficulty with tracer delivery.

### Clinical evaluations

Participants underwent clinical evaluation, including motor assessment with the Movement Disorder Society-sponsored Unified Parkinson’s Disease Rating Scale Part III (MDS-UPDRS III), cognitive testing (MoCA; Dementia Rating Scale-2, DRS-2) and Beck Depression Inventory (BDI). The clinical evaluations were performed in the off state, after withdrawal of dopaminergic medication for 12-to-48 h, depending on the drug.

### Tracer production

[^11^C]CO2 is produced by proton irradiation with nitrogen target gas containing 5% O_2_ using the ^14^N(p, α)11C reaction with in-target formation of CO_2_ by concomitant reaction with O_2_. The irradiation is carried out on the TR13 cyclotron. After the irradiation time, [^11^C]CO2 is then converted to [^11^C]MeI using the GE Tracerlab FXC Pro synthesis module. The [^11^C]MeI is transferred to a DMF solution of the precursor (5-((1E,3E)-4-(6-((tert-butlydimethylsilyl)oxy)benzo[d]thiazol-2- yl)buta-1,3-dien-1-yl)pyridine-2-amine) and sodium hydride solution and kept at 110 °C for 4.5 min. Then the reaction is cooled to 70 °C, and 0.4 mL of water is added to the reaction mixture. The reaction is cooled further to 50 °C for 1.5 min. The reaction mixture is purified using HPLC. The product peak is collected directly into the product vessel and mixed with 5 mL of 0.9% saline. The final formulation is then passed through a 0.22 µm filter into a sterile vial and released for QC analysis.

### Image acquisition and analysis

All participants underwent anatomical MRI and PET scans with 2-((1E,3E)-4-(6-([^11^C]methylamino)pyridin-3-yl)buta-1,3-dienyl) benzo[d]thiazol-6-ol ([^11^C]PBB3). All but two of the PD participants (1 CN, 1 CI) also underwent PET with [^11^C] (+)dihydrotetrabenazine ([^11^C](+)DTBZ), a vesicular monoamine transporter 2 marker, to measure dopaminergic integrity. Nine randomly selected CN-PD patients (all with high or mixed affinity binding to TSPO based on testing for the rs6791 polymorphism^41^) underwent PET scan with [^11^C]PBR28, a second generation TSPO tracer.

PET scans were performed on a Siemens High Resolution Research Tomograph (HRRT, Knoxville, TN) with a spatial resolution of (2.5 mm)^3^. A transmission scan was performed using a ^137^Cs source for attenuation correction. Subjects were positioned using external lasers aligning the gantry with the inferior orbital-external meatal line, and custom-fitted thermoplastic masks were applied to minimize head movement. Because PBB3 is subject to degradation by light, the bolus was injected with photoprotection. Emission data were collected for 70 minutes. Each subject also underwent a structural (T1-weighted sequence) brain MRI on a 3.0 Tesla Philips Achieva whole-body MRI scanner (Philips Healthcare, Amsterdam, The Netherlands). [^11^C]PBR28 data were dynamically acquired over 90 min.

PET images were coregistered to the participant’s anatomical MRI. The MRI images were resized to PET voxel size from (1 mm)^3^ to (1.22 mm)^3^ (voxel size of reconstructed images). The dynamic images were first frame-to-frame realigned. PET images were coregistered to the subject’s anatomical MRI, which were reoriented to the anterior-posterior commissural line. MRI images were then coregistered to the Montreal Neurological Institute (MNI) standard coordinate frame. For [^11^C]PBB3, geometric regions of interest (ROIs) were defined using PMOD’s PNEURO tool in MNI space and then all in one step inverse-transformed into the subject’s original PET space and placed on the PET images. All transformations were performed with Statistical Parametric Mapping software (SPM12; Wellcome Trust Center for Neuroimaging, London, UK). Time-activity curves were extracted for each ROI. Non-displaceable binding potential (BP_ND_) values were calculated using the Logan reference tissue method^42^ with cerebellar white matter as reference region. Reference tissue models provide an excellent estimate of BP_ND_ despite the presence of radiolabeled metabolites that cross the blood-brain barrier^43^. Considering the possible effect of age and PD neuropathology on SN volume, we used the region-based voxel-wise partial-volume correction from PMOD^44^. [^11^C]PBR28 scans were analyzed using a similar approach to determine standardized uptake values (SUV) evaluated over 60–90 min post-injection. For [^11^C](+)DTBZ, ROI placement was limited to the striata; one ROI on each caudate and three on each putamen (i.e., anterior, middle and posterior putamen). The tissue input Logan method with an occipital cortex reference region was used to determine DTBZ BP_ND_ values. The team performing the image analysis was blind to the diagnosis of the participants.

### PBB3 binding in dopaminergic regions

We calculated the regional [^11^C]PBB3 BP_ND_ in the SN, the head of caudate nucleus and the putamen. To study putaminal involvement in greater topological detail, three different circular ROIs were visually placed in the anterior, middle, and posterior part of the putamen. We compared the average BP_ND_ of both hemispheres in HC vs PD for each of those five regions. As PD is typically asymmetric, we also separately compared [^11^C]PBB3 average binding in HC with the more and the less affected hemisphere of the CN-PD cohort. The more affected side was defined as the brain hemisphere contralateral to the more affected body side. We also repeated the analysis in an exploratory fashion after excluding CI-PD subjects, to eliminate the confound of possible tau deposition related to cognitive impairment.

We also studied the correlation between [^11^C]PBB3 binding and (i) disease duration in the PD cohort, adding age as a covariate and (ii) [^11^C](+)DTBZ binding (as a measure of dopaminergic innervation) in the striatal subregions.

Finally, we investigated a possible age dependence of [^11^C]PBB3 binding in these ROIs. We used linear regression analysis to model BP_ND_ at different ages in CN-PD and HC, with disease duration added as covariate in the PD group.

### PBB3 binding in other ROIs

As an additional exploratory analysis, we evaluated CN-PD [^11^C]PBB3 BP_ND_ in the following cortical and subcortical regions that may be implicated in PD: amygdala, hippocampus, anterior cingulate, middle frontal gyrus, temporal cortex, occipital cortex, parahippocampal gyrus (including the entorhinal cortex), pons and thalamus.

### PBB3 binding and cognition

We compared [^11^C]PBB3 BP_ND_ between HC, CN-PD and CI-PD in cortical and subcortical regions. We further investigated the relationship between [^11^C]PBB3 BP_ND_ and DRS-2 score in all PD participants (including both CN-PD and CI-PD).

### PBB3 and PBR28 binding correlation

As protein misfolding in neurodegeneration may be associated with an inflammatory response, and as many tau tracers may bind to monoamine oxidase (MAO) isoforms, which are associated with glial inflammatory reactions, we studied the correlation between [^11^C]PBB3 and [^11^C]PBR28 binding *across subjects* in a subset of PD patients. Given the susceptibility of PBR28 binding to TSPO rs6971 polymorphism^45^, we restricted our primary analysis to high affinity binders (*n* = 6/9 PD patients studied), excluding low and medium affinity binders (not enough signal and not enough subjects respectively). In addition, given the possible bias of [^11^C]PBR28 SUV compared to plasma input measures^46^, a *within-patient* rank-based comparison was performed for all 13 brain regions in which values from both tracers had been obtained. We repeated this analysis also including the three medium affinity binders, as within-individual ranks should not be impacted by the rs6971 polymorphism. Finally, as a post-hoc analysis, we studied the relationship between the individual rank correlations and disease duration.

### Statistical analysis

Due to non-normality of distribution, baseline characteristics and [^11^C]PBB3 BP_ND_ comparisons were performed using non-parametric two-tailed tests (Mann–Whitney *U* test for two-group comparisons, Kruskal–Wallis for three-group comparisons for continuous variables and Fisher’s exact test for categorical variables). The relationships between [^11^C]PBB3 BP_ND_ and [^11^C]PBR28 SUV and between [^11^C]PBB3 and [^11^C](+)DTBZ binding were studied using Spearman’s correlation and the relationship of [^11^C]PBB3 with clinical outcomes was studied using a forward stepwise linear regression analysis including age and BDI as covariates for cognitive outcomes, to account for possible confounding effects. Significant results of linear regression analysis were visually checked for normal distribution of residuals. The missing (at random) data in our study were handled using complete case analysis. The level α was set for all comparisons to *p* < 0.05. Results were adjusted for multiple comparisons using the Bonferroni method and unless otherwise specified, all reported *p*-values are appropriately adjusted. All statistical analyses were performed using R 4.2.1 (https://www.R-project.org).

### Reporting summary

Further information on research design is available in the Nature Research Reporting Summary linked to this article.

### Supplementary information

Supplementary material

Reporting Summary

## Data Availability

Anonymized data will be made available to qualified academic investigator upon written request.
