# Supplementary material for: Misfolded protein deposits in Parkinson’s disease and Parkinson’s disease-related cognitive impairment, a [11C]PBB3 study
Source: NPJ Parkinsons Dis. 2024 May 3;10:96. doi: 10.1038/s41531-024-00708-z (PMC11068893; doi:10.1038/s41531-024-00708-z)
Supplement: Supplementary file 1 — Supplementary material [file 41531_2024_708_MOESM1_ESM.pdf]

Supplementary materials

Table of Contents

Supplementary Figures......1

Supplementary Figure 1. Correlation between rank [11C] PBB3 and rank [11C] PBR28 binding at different disease durations. 1

Supplementary tables......2

Supplementary Table 1. Comparison of [11C]PBB3 binding in dopaminergic regions in cognitively normal PD subjects and healthy controls 2

Supplementary Table 2. Within-subject correlation of rank [11C] PBR28 and [11C] PBB3 binding across brain regions in PD subjects. 2

Supplementary Figures

Supplementary Figure 1. Correlation between rank [11C] PBB3 and rank [11C] PBR28 binding at different disease durations.

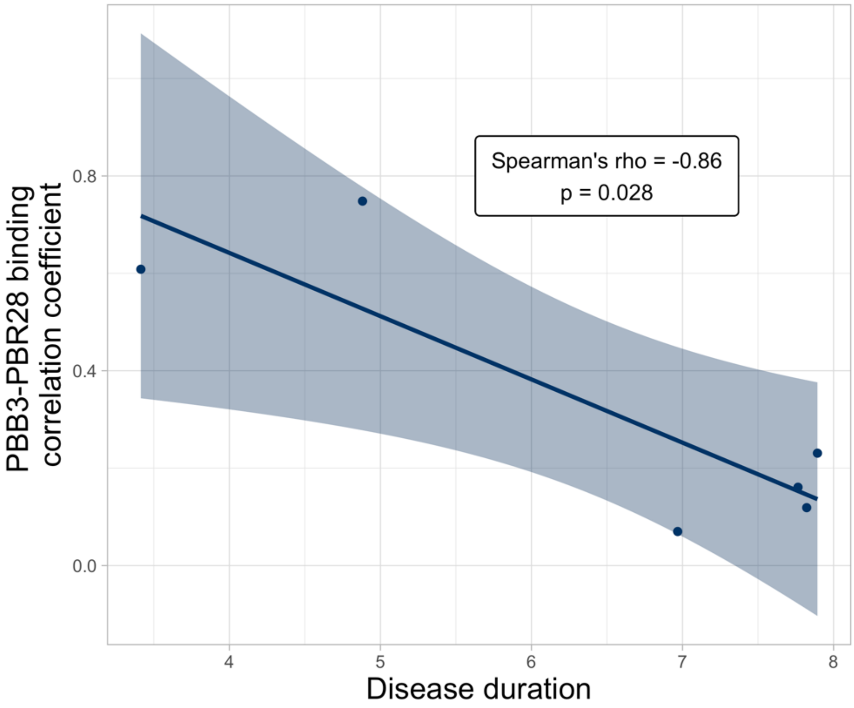

Correlation between the individual [11C] PBB3 vs [11C] PBR28 binding ranked correlation coefficient (including 13 brain regions) and disease duration.

Supplementary tables

Supplementary Table 1. Comparison of [^{11}C]PBB3 binding in dopaminergic regions in cognitively normal PD subjects and healthy controls

	Group		
	Healthy controls (n = 10)	PD subjects (n = 32)	Multiple comparisons adjusted *p* value
Substantia nigra	0.24 [0.15, 0.26]	0.29 [0.20, 0.34]	0.845
Caudate nucleus	0.29 [0.20, 0.35]	0.28 [0.19, 0.36]	1
Anterior putamen	0.39 [0.34, 0.52]	0.48 [0.39, 0.54]	1
Middle putamen	0.44 [0.38, 0.47]	0.55 [0.42, 0.67]	0,595
Posterior putamen	0.29 [0.28, 0.33]	0.44 [0.36, 0.52]	**0.02**

CN-PD, Cognitively normal Parkinson's disease subjects.
Variables are represented as "median [IQR]"

Supplementary Table 2. Within-subject correlation of rank [^{11}C] PBR28 and [^{11}C] PBB3 binding across brain regions in PD subjects.

Participant	Disease duration	Spearman Rho	*p* value
1	7.0	0.07	0.83
2	4.9	0.75	**0.01**
3	7.8	0.12	0.72
4	7.9	0.23	0.47
5	7.8	0.16	0.62
6	3.4	0.61	**0.04**
